# Impact of introducing capacity-based mental health legislation on the use of community treatment orders in Norway: case registry study

**DOI:** 10.1192/bjo.2021.1073

**Published:** 2022-01-07

**Authors:** Georg Høyer, Olav Nyttingnes, Jorun Rugkåsa, Ekaterina Sharashova, Tone Breines Simonsen, Anne Høye, Henriette Riley

**Affiliations:** Division of Mental Health and Substance Abuse, University Hospital of North Norway, Norway; and Department of Community Medicine, UiT The Arctic University of Norway, Norway; Health Services Research Unit, Akershus University Hospital, Norway; and R&D Department, Division of Mental Health Services, Akershus University Hospital, Norway; Health Services Research Unit, Akershus University Hospital, Norway; and Centre for Care Research, University of South-Eastern Norway, Norway; Department of Community Medicine, UiT The Arctic University of Norway, Norway; Health Services Research Unit, Akershus University Hospital, Norway; Department of Clinical Medicine, UiT The Arctic University of Norway, Norway; and Division of Mental Health and Substance Abuse, University Hospital of North Norway, Norway; Division of Mental Health and Substance Abuse, University Hospital of North Norway, Norway; and Department of Health and Care Sciences, Faculty of Health Sciences, UiT The Arctic University of Norway, Norway

**Keywords:** Capacity-based mental health law, community treatment orders, compulsion, mental health legislation

## Abstract

**Background:**

In 2017, a capacity-based criterion was added to the Norwegian Mental Health Act, stating that those with capacity to consent to treatment cannot be subjected to involuntary care unless there is risk to themselves or others. This was expected to reduce incidence and prevalence rates, and the duration of episodes of involuntary care, in particular regarding community treatment orders (CTOs).

**Aims:**

The aim was to investigate whether the capacity-based criterion had the expected impact on the use of CTOs.

**Method:**

This retrospective case register study included two catchment areas serving 16% of the Norwegian population (aged ≥18). In total, 760 patients subject to 921 CTOs between 1 January 2015 and 31 December 2019 were included to compare the use of CTOs 2 years before and 2 years after the legal reform.

**Results:**

CTO incidence rates and duration did not change after the reform, whereas prevalence rates were significantly reduced. This was explained by a sharp increase in termination of CTOs in the year of the reform, after which it reduced and settled on a slightly higher leven than before the reform. We found an unexpected significant increase in the use of involuntary treatment orders for patients on CTOs after the reform.

**Conclusions:**

The expected impact on CTO use of introducing a capacity-based criterion in the Norwegian Mental Health Act was not confirmed by our study. Given the existing challenges related to defining and assessing decision-making capacity, studies examining the validity of capacity assessments and their impact on the use of coercion in clinical practice are urgently needed.

## Background

Over recent years there have been increased efforts in many jurisdictions to reduce the use of coercion in mental health services.^1^ In this context, the development of the United Nations Convention on the Rights of Persons with Disabilities (CRPD) in 2006 has been described as a paradigm shift in the treatment of people with mental disabilities.^2–4^ At the heart of the convention is a shift from diagnosis-based criteria for involuntary care and treatment, to legislation founded on patient autonomy. Several European and Australian jurisdictions have introduced capacity-based mental health legislation in order to strengthen patient autonomy.^5,6^ On 1 September 2017, an amendment to the Norwegian Mental Health Act (NMHA) came into force, prescribing that only people who lack decision-making capacity can be subjected to involuntary care or treatment, unless there is an imminent risk to the patient's life or the life and health of others.^7^ The wording of the capacity criterion in the NMHA is brief, stating that compulsion is only permissible if ʻthe patient lacks capacity to consent', with reference to the Patients’ Rights Act (see the Appendix). The capacity criterion in the latter states that ʻcompetence to give consent may cease to apply wholly or partly if the patient, on account of a physical or mental disorder, senile dementia or mental retardation, is clearly incapable of understanding what the consent entails'.^8^ The Ministry of Health expected that by raising the threshold for coercion the use of involuntary care and treatment would reduce, in particular the use of community treatment orders (CTOs), which are issued as patients leave hospital and presumably when they are more likely to have capacity. It was also expected that the duration of involuntary care episodes would reduce, as well as the number and duration of involuntary treatment orders (which under Norwegian law are required as an additional order to the involuntary placement order) issued to insist on medication or other forms of treatment. It was further assumed that the reform would strengthen the autonomy and improve the legal safeguards of people with mental disorders, thus improve compliance with the CRPD.^9,10^

The criteria for involuntary placement and treatment in Norway are the same for CTOs and in-patient care. In addition to the newly introduced capacity criterion, the NMHA requires that the patient has a severe mental disorder, needs treatment and/or represents a risk to their own life or the life and health of others (Appendix).

All involuntary placement orders, including CTOs, must be reviewed by the responsible clinician (a psychologist or psychiatrist) every third month, and this includes a review of the patients’ capacity. Prolongations for more than 1 year need authorisation by a mental health review board (‘control commissions’). Decisions made by the control commissions can, in turn, be appealed to the courts. The NMHA permits CTOs to be made without a prior involuntary in-patient period, although clinicians rarely use this option.^11,12^ Following the legal change, the capacity to consent criterion becomes irrelevant if the dangerousness criterion is fulfilled, and this raised the authorities’ concern that more patients would now be assessed as dangerous.

## Aims

Despite considerable theoretical interest and debate,^6^ we have not been able to identify any empirical studies on the impact of capacity-based mental health legislation. To ascertain any such impact on the use of CTOs, the present study compares the use of CTOs 2 years before and 2 years after the capacity to consent criterion came into force in Norway. Specifically, we investigate whether there were any changes in (a) the incidence and prevalence rates of CTOs, (b) the duration of CTOs, (c) the use of involuntary treatment orders, and (d) clinicians’ justification for using CTOs.

## Method

### Study setting and design

The study is a retrospective case register study on the use of CTO in two Norwegian regions in 2015–2019. The two study sites, the University Hospital of North Norway and Akershus University Hospital, are located in two very different geographical regions in Norway,^11,12^ that represent both urban and rural areas and serve a combined population of 678 214 aged 18 years or above (2017 census figures), which corresponds to 16% of the total Norwegian adult population.

We decided to exclude the year the legal reform came into force (2017) in the pre/post analysis of possible effects, on the presumption that clinicians needed time to prepare for and adapt to a substantial change in the legal framework regulating involuntary care and treatment. Therefore, the year of the reform would likely not be representative of any sustained changes to practice.^13^ In reporting our findings, we follow the STROBE checklist.

### Data sources

National statistics on the use of CTOs do not include their end-points, and could not be used to answer our research questions. All data was collected from patients’ individual electronic medical records between 1 January 2015 and 31 December 2019. Eligible patients were identified through the ʻDIPS' electronic information and patient records system, which is used by most hospitals in Norway. We cooperated closely with administrative staff working with this system to identify all potential patients. A detailed registration form and coding guide was created to ensure consistency across sites, and we held regular team meetings to resolve any uncertainties in how to interpret data. The form was constructed in REDCap (Research Electronic Data Capture) and all data were manually entered there before being converted into a data-set and imported into SAS and SPSS. Ten cases were piloted, which resulted in minor adjustments to the registration form. Data on population size was obtained from Statistics Norway.^14^

### Study participants

At both study sites, all patients aged 18 years or above who were subjected to CTO at least once in the study period, were included. Some services appeared to use CTOs to facilitate patient transfers from one in-patient service to another, or for short-term in-patient stays in other, non-psychiatric facilities (such as somatic care). Accordingly, CTOs lasting less than 7 days were excluded, as we considered such very short-term CTOs not to be about psychiatric community treatment. Patients who were placed on a CTO but lived and received community care outside the catchment areas were also excluded. For those who moved out of the catchment areas while on a CTO, we set the date of their move as their end-point. For patients who died during the study period, their end-point was their date of death. All analyses concern new CTOs, except for the point-prevalence rate calculation, which included CTOs in place on 1 January 2015.

### Statistical analysis

Analyses were performed using SAS for Windows v. 9.4 and SPSS for Windows v. 26. Descriptive statistics are presented as means (s.d.) or medians (first and third quartiles) for continuous variables, and numbers (percentages) for categorical variables. To compare all new CTOs in the period 1 January 2015–31 December 2016 with all new CTOs between 1 January 2018 and 31 December 2019, we used independent samples *t*-test, Moods median test and Pearson's chi-squared test. As the pre/post groups were not fully independent (62 of 631 individuals (9.8%) had CTOs in both periods) we also used linear mixed models and generalised estimating equations to test for between-group differences and adjust for correlated within-subject errors. This did not alter the pattern of results (expressed in *P*-values) and therefore these results are not shown. We expected some CTOs to last longer than the study period, and thus extend across pre and post stages. For the analysis of CTO duration, we therefore set 31 December 2016 as the end-point for all CTOs that started in 2015–16 but were not terminated before that date. For CTOs commencing in 2018–2019, we set 31 December 2019 as the end-point if they were not terminated before. We conducted a *post hoc* analysis to explore possible changes in the duration of CTOs in more detail, based on their year of initiation. Duration of all CTOs was measured from their initiation, with 31 December of the subsequent year as the end-point unless they ended before (new CTOs in 2015 were followed until the end of 2016 etc.). We estimated a mixed-effects model of CTO duration measured this way, with initiation year as the independent variable and a random intercept for patients.

Incidence rates and point-prevalence rates were calculated per 100 000 population (18 years of age or above) for 3-month periods and for each study year from 2015 to 2019. Point-prevalence rates were calculated based on the number of CTOs on the last day of the corresponding period. Point-prevalence rate ratio (95% confidence interval, *P*-value) was calculated to compare point-prevalence rate per 31 December 2019 and per 31 December 2016.

CTO termination rates per quarter were calculated as number of terminations per 100 CTOs. To test for change in the rate of CTO terminations, we conducted an independent samples *t*-test of rates in the eight quarters before and eight quarters after 2017, using robust standard errors. As 76 of the 627 patients with CTO terminations had more than one during the study period, this should be interpreted cautiously. As there are geographical variation in the use of compulsion,^10–12^ we compared patient characteristics and all outcomes between the two sites using Pearson's chi-squared and Mann–Whitney U-tests as appropriate.

### Ethics

The authors assert that all procedures contributing to this work comply with the ethical standards of the relevant national research frameworks and with the Helsinki Declaration of 1975, as revised in 2008. All procedures involving human patients were approved by the Regional Committee for Medical and Health Research Ethics, Region North (REC North, ref: 2010/2268). As completeness of data was crucial to ensure accurate figures for the incidence, prevalence and duration of CTO, the committee granted access to medical files without consent from individual patients. All data were de-identified before being stored and used in the analysis.

## Results

### Participants

We identified 760 patients who were subjected to a total of 921 new CTOs in the study period. The majority (625) had one CTO episode, 113 had two, 18 had three and 4 had four. There were no changes in the distribution of age, gender, diagnosis or concern over substance use before and after the reform, or between the two study sites (results for site comparisons are not shown).

The mean age was 42.4 years (s.d. = 16.0), and the gender distribution was 43.2% females and 56.8% males. A total of 77.9% were diagnosed with a schizophrenia spectrum disorder (ICD-10 F20–29^15^) and 14.6% with an affective disorder (ICD-10 F30–39). The remaining 57 patients (7.5%) were diagnosed with a range of other disorders (such as organic, substance misuse, personality disorders and eating disorders), the symptoms of which may be so disabling as to meet the legal criteria of severe mental disorder. Concerns over drug or alcohol use were noted in the records of 35.1% of patients (Table 1).

**Table 1:** Demographic and clinical characteristics of the 760 patients placed on community treatment orders (CTOs) during the study period^a^

Variable	2015–2016 (*n* = 345)	2017 (*n* = 184)	2018–2019 (*n* = 348)	Total (*n* = 760)	*P*^b^
Age in years, mean (s.d.)	42.8 (15.3)	42.8 (17.0)	41.5 (15.6)	42.4 (16.0)	0.29
Gender, *n* (%)					
Female	157 (45.5)	80 (43.5)	147 (42.2)	328 (43.2)	0.39
Male	188 (54.5)	104 (56.5)	201 (57.8)	432 (56.8)	
ICD-10 diagnosis, *n* (%)					
F20–F29	264 (76.5)	149 (81.0)	278 (79.9)	592 (77.9)	0.54
F30–F39	56 (16.2)	22 (12.0)	50 (14.4)	111 (14.6)	
Other	25 (7.2)	13 (7.1)	20 (5.7)	57 (7.5)	
Concern over substance use recorded, *n* (%)					
Yes	115 (33.3)	75 (40.8)	122 (35.1)	267 (35.1)	0.63
The number of CTO episodes experienced by individual patients, *n* (%)					
1	328 (95.1)	182 (98.9)	325 (93.4)	625 (82.2)	0.34^c^
2	17 (4.9)	2 (1.1)	22 (6.3)	113 (14.9)	–
3	–	–	1 (0.3)	18 (2.4)	–
4	–	–	–	4 (0.5)	–

a. Data are for all variables, except for number of CTOs, are for a patient's first CTO in each period. Nine patients were placed on a CTO in all three periods. In total, 53 patients were placed on a CTO in both 2015–2016 and 2018–2019, 17 in both 2015–2016 and 2017, and 29 in both 2017 and 2018–2019.

b. *P*-values for differences between 2015–2016 and 2018–2019, using independent samples *t*-test for age and chi-square for the remaining variables.

c. χ² for 1 versus >1 CTO.

### Changes in the rates of CTO incidence, point prevalence and termination

**Fig. 1:**
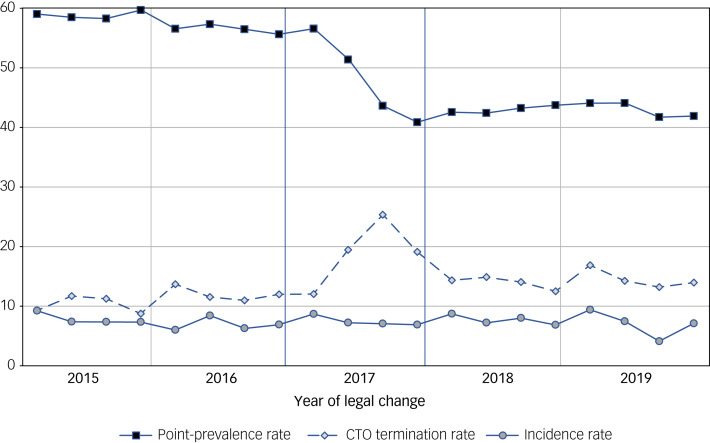
Quarterly incidence, point-prevalence and termination rates of community treatment orders (CTOs) in two Norwegian hospital areas. Point-prevalence and incidence rates are CTOs per 100 000 population (≥18 years). CTO termination are number per 100 CTOs. The legal reform was passed in April 2017 and took effect 1 September that year.

Quarterly rates of incidence, point prevalence and termination of CTOs across the period are depicted in Fig. 1.

Incidence rates were remarkably stable over the whole study period, 2017 included, varying between a quarterly median rate of 7.4 (Q1–Q3 6.5–8.2) per 100 000 adult capita in the period before the reform, 7.2 (Q1–Q3 7.0–8.5) in the year of the reform and 7.5 (Q1–Q3 7.1–8.5) after. Point-prevalence rates were significantly reduced from a median of 57.8 (Q1–Q3 56.5–58.8) before the reform to a median of 43.0 (Q1–Q3 42.24–44.11) after (0.77, 95% CI 0.66–0.91, *P* < 0.001).

CTO termination rates were stable in the period before the reform with a median of 11.4 terminations per 100 CTOs per quarter (Q1–Q3 9.6–11.9). In 2017, there was a sharp rise from the first to the third quarter, from 12.0 to 25.3 per 100 CTOs (*P* < 0.01). After September 2017 (when the law took effect), there was also a sharp decline, until the first quarter of 2018, when termination rates stabilised at a 24% higher level (median 14.1, Q1–Q3 13.3–14.8) for 2018–2019 as compared with 2015–2016. An independent samples *t*-test of termination rates in the eight quarters before and eight quarters after 2017 showed that the increase in termination rates was significant (*P* = 0.001).

### Changes in the duration of CTOs

As shown in Table 2, we found a small reduction in the median duration of CTO from 175 days (Q1–Q3 76.0–353.5) to 158 days (Q1–Q3 65.0–326.5) before and after the reform, but this reduction was not statistically significant (*P* = 0.53). In the exploratory analysis of CTO duration by year of initiation, we found a significant reduction in duration from 2015 to 2016 (323 and 268 days, respectively, *P* < 0.011) which continued in 2017 (254 days *P* < 0.001), but from 2018 the duration of CTOs increased again (306 days) and was not significantly different from that in 2015 (*P* = 0.39).

**Table 2:** Changes in duration of community treatment orders (CTOs), use of involuntary treatment orders and in justifications for establishing and terminating CTOs after the introduction of the capacity to consent criterion

Variable	New CTOs in each time period				
	2015–2016 (*n* = 362)	2017 (*n* = 186)	2018–2019 (*n* = 373)	Total (*n* = 921)	*P*^a^
Duration of CTOs in days, median (Q1–Q3)^b^	175 (76−353.5)	174 (77−395.5)	158 (65−326.5)	168 (70–354)	0.53
Duration of CTOs in days, mean (s.d.)^b^	235.3 (197.3)	250.0 (198.1)	222.7 (189.5)	233.2 (194.4)	0.38
Having an involuntary treatment order in addition to the CTO, yes: *n* (%)	184 (50.8)	119 (64.0)	259 (69.4)	562 (61.0)	<0.001
Additional criteria justifying the CTO^c^					
The need for treatment on its own, *n* (%)	318 (87.8)	160 (86.0)	322 (86.3)	800 (86.9)	0.54
The dangerousness criterion on its own or combined, *n* (%)	41 (11.3)	25 (13.4)	50 (13.4)	116 (12.6)	0.39
Not documented, *n* (%)	3 (0.8)	1 (0.5)	1 (0.3)	5 (0.5)	0.30
Recorded reason for terminating the CTO^d^					
Need for treatment criterion no longer applies, *n* (%)	4 (1.1)	7 (3.8)	12 (3.2)	23 (2.5)	0.50
Dangerousness criterion no longer applies, *n* (%)	0 (0.0)	0 (0.0)	0 (0.0)	0 (0.0)	−
Patient cooperates voluntarily, *n* (%)	141 (39.0)	113 (60.8)	148 (39.7)	402 (43.6)	0.84
Patient has capacity to consent, *n* (%)	3 (0.8)	67 (36.0)	118 (31.6)	188 (20.4)	<0.001
CTO lifted by control commission or court, *n* (%)	9 (2.5)	7 (3.8)	24 (6.4)	40 (4.3)	0.01
Patient died, *n* (%)	2 (0.6)	4 (2.2)	6 (1.6)	12 (1.3)	0.17
CTO not terminated by end of period, *n* (%)	178 (49.2)	41 (22.0)	164 (44.0)	383 (41.6)	0.15

a. *P*-values calculated for differences between 2015–2016 and 2018–2019 using chi-squared and Moods median test and *t*-test for independent samples as appropriate.

b. To ensure comparability between the pre and post reform periods, CTOs that started in 2015–2016 still in place on 31 December 2016 were given this date as their end-point, and CTOs that started in 2018–2019 still in place on 31 December 2019 were given this date as end-point. For CTOs that started in 2017, 31 December 2018 was set as end-point if not terminated before.

c. Additional to the criteria of (a) the presence of a severe mental disorder (a psychotic disorder, or other disorders with symptom severity equally disabling as a psychotic state) and, from 1 September 2017 (b) the absence of capacity to consent.

d. More than one reason for terminating a CTO could be recorded. To ensure comparability between the pre and post reform periods, we only included CTO terminations that happened within the same time periods for which duration of CTOs was calculated.

### Changes in the use of involuntary treatment orders

We found a significant increase in the number of involuntary treatment orders for patients with a CTO, from 50.8% before to 69.4% after the reform (*P* < 0.001) (Table 2).

### Changes in the justifications for establishing and terminating CTOs

We found no significant changes in the justifications for new CTO orders after the legal reform, as also shown in Table 2. For the additional legal criteria required for the CTO to be valid, the use of the need for treatment criterion on its own changed from 87.8% of the cases before to 86.3% after the reform (*P* = 0.54). The use of the dangerousness criterion (on its own or in combination with the need for treatment criteria) increased from 11.3% to 13.4% of cases pre–post 2017 (*P* = 0.39). The dangerousness criteria on its own was used only in 1–1.1% of cases over the full study period.

There were significant changes in the stated reasons for terminating CTOs. After the reform, 31.6% of terminations were because of the patient having decision-making capacity, compared with 0.8% before (*P* < 0.001). There was also a significant increase in the number of CTOs lifted by review boards (control commissions) and courts after the legal reform (2.5% to 6.4%, *P* = 0.01) (Table 2). The was no change in the number of CTOs that were terminated because the patient cooperated on a voluntary basis.

We compared all outcomes across the two study sites. No statistically significant differences were found and these results are therefore not shown.

## Discussion

The aim of the study was to investigate the impact of introducing a capacity-based criterion into the NMHA on the use of CTOs. Given that those on a CTO generally can be expected to function at a higher level than their in-patient counterparts, it was assumed that they would be more likely to be assessed competent to consent to, or refuse, mental healthcare and treatment. Accordingly, we assumed that the new capacity criterion would affect CTOs to a larger degree than could be expected for those in in-patient involuntary care.

### Impact of the legal reform on prevalence rates, incidence rates and duration of CTOs

After the legal change, those deemed to have decision-making capacity can no longer be placed on a CTO, and the ‘pool’ of eligible CTO candidates should therefore be smaller. Yet, despite a reduction in point-prevalence rates, we did not find any evidence of reductions in incidence rates of CTOs, and only a small, insignificant reduction in their duration. We did not observe any change in the use of the need for treatment or the dangerousness criteria as reasons for why the CTO was necessary (Table 2). We thus believe that the patients subjected to CTO did not differ before and after the legal reform. This raises the question of why the changes, as expected by the Ministry of Health, did not materialise.

It has previously been suggested that there is an implicit consensus among stakeholders on which patients needed to be treated in hospital and that this was refectory to legal frameworks.^13^ Another possible explanation could be that clinicians before the legal reform already issued involuntary care and treatment orders only for those who were unable to give a valid consent. If this is the case, focus should be directed towards how clinicians assess patients’ capacity to consent. Given many identified challenges related to defining and assessing decision-making capacity,^16,17^ and the lack of empirical evidence on how clinicians actually assess it, studies validating capacity assessments in everyday clinical practice are needed. Future research should include entire populations of patients that are considered for, of referred to, CTO placement, including the rationale and outcome of referrals and capacity assessments at different points of the CTO placement.

The legal reform was also expected to reduce the rates of involuntary in-patient care,^9,10^ but national figures for incidence rates for 2018 and 2019 show the same pattern as we found for CTOs, i.e. they remained unchanged.^18^ This is another indication that the introduction of the capacity to consent criterion had little effect on the use of coercion across service levels.

Our data confirm that 2017 was a ‘different’ year (Fig. 1). The significant reduction in point-prevalence rate and rise in termination rates in the first 9 months of 2017, after which the termination rate stabilised, suggest, in our opinion, that with the legal change on the horizon, clinicians might have ‘weeded out’ a number of long-term CTOs for patients they expected would be assessed as having decision-making capacity. We have previously shown how CTO periods for some patients in Norwegian services almost routinely are prolonged over many years.^11^ The shorter durations of CTOs during 2017 compared with the 2 years before and after is likely also a result of this ‘weeding out’ process. We are not aware of other studies that have identified and discussed such ‘weeding out’ effects in relation to legal reforms, and it is not clear whether our finding corresponds to clinicians’ reactions to legal reforms more generally.

The concern expressed by Norwegian health authorities that more patients would be assessed as dangerous to themselves or others to ‘evade’ the new capacity to consent criterion, was not confirmed by our data, as we found no increase in the use of the dangerousness criterion on its own or in combination with other criteria. A likely explanation why this feared use of the dangerousness criterion did not manifest might be that clinicians are aware of possible serious negative consequences for anyone labelled as dangerous, and thus would not classify someone as such for opportunistic reasons.

### Increased use of involuntary treatment orders

The significant increase in the rate of involuntary treatment orders in conjunction with CTOs after the legal change was surprising and contrary to the health authorities’ expectations. We suspect that this likely reflects that the group of patients on CTOs without an involuntary treatment order who neither consented nor objected to treatment were more frequently subjected to a formal treatment order after the legal reform. This would be in line with circulars issued between 2017 and 2021 by the Norwegian Directorate of Health. With reference to the new criterion in the NMHA, these instruct clinicians to make formal commitment and treatment orders for all patients whose consent to care and treatment is considered invalid because of their lack of capacity. One reason for this instruction was that without a formal order, patients are deprived of the legal safeguards that comes with involuntary care and treatment orders. The magnitude of the issue of the so-called ‘informal patients’ without capacity, that is patients who neither give a valid consent nor object to care and treatment, was suggested in relation to the 1997 ‘Bournewood case’ in the UK. It was estimated that if all patients in this category should be formally committed, the prevalence of formally detained patients in psychiatric institutions in the UK at the time would increase from 13 000 to 35 000.^19^ We are not aware of other studies that address the problem of the (unknown) number of informal patients who are *de facto* deprived of their liberty and subjected to care and treatment against their will. It is probable that the up to 20-fold variation in civil commitment rates between European countries^5^ at least partly can be explained by variations in the numbers of informal patients who *de facto* are subjected to treatment without a valid consent. We believe that the observed increase in involuntary treatment orders found in our study does not reflect an increase in the number of patients who *de facto* are involuntary treated. The finding does rather indicate that clinicians have complied with the instructions from the Norwegian health authorities to issue involuntary treatment orders for people who lack decision-making competence, even if they do not resist the actual treatment.

### Reasons for termination of CTOs and acknowledgement of legal capacity

The observed increase after the reform of CTOs that were lifted because the patient was found to have decision-making capacity might have different explanations. One possible explanation could be that clinicians, who were all offered training in capacity assessments in the lead up to the reform, focused on and were better qualified to recognise patients’ legal capacity than before. However, we believe there is another more likely explanation: before the reform clinicians were not equally concerned whether the patients had decision-making competence or not if they voluntarily complied with treatment, whereas after the reform clinicians were obliged to end involuntary care and treatment for patients who were assessed to have capacity, even if they did not object to being subjected to a CTO and complied without resistance.

We also found a significant increase in the number of CTOs lifted by control commissions and the court after the reform. Even if the numbers were small (9 cases before the reform and 24 cases after) this raises questions about the role of complaints and review mechanisms in relation to implementation of new legal standards, which as far as we have observed is another under-researched area.

### The NMHA and compliance with the CRPD

The Norwegian Ministry of Health argued that the capacity-based criterion would improve the compliance of the NMHA with the CRPD. When the CRPD was adopted by the United Nations in 2006, it was referred to as a paradigm shift concerning the rights and autonomy of people with disabilities, including those with mental disorders.^2–4^ Initiatives such as shared or assisted decision-making, combined with capacity-based legislation, would enable more people with mental disabilities to make autonomous decisions and thus reduce the use of coercive interventions. The CRPD, however, goes further in its ambition to protect patient autonomy. State parties to the convention are obliged to put in place effective safeguards that enable patients’ exercise of legal capacity to ensure that their rights, will and preferences are respected. An anti-discriminatory principle underpinning the CRPD states that: ʻperceived or actual deficits in mental capacity must not be used as justification for denying legal capacity'.^20^ Despite the introduction of the competence to consent criterion in the NMHA, and the safeguards that comes with a formal order, for Norway to increase its compliance with the CRPD, mechanisms for enhancing patients’ legal capacity and/or respecting their will and preferences ought to be found.

There are two additional elements of the NMHA that prevent compliance, and, according to the principle outlined above, render it discriminatory. First, the competence to consent criterion does not apply if the patient is considered to be dangerous, and second, the primary criterion for involuntary interventions is based on diagnoses, i.e. that the person has a severe mental disorder. The debate on the implications of the CRPD has been heated since its adoption, and especially so after the CRPD committee published their interpretation of article 12 in 2014.^20^ The committee stated that all people with disabilities who can express their will and preferences, including those with mental disorders who might be dangerous to themselves or others, should be considered to have legal capacity to make decisions. This sparked arguments for and against such a position.^3,4,21,22^ Combined with the stance that any reference to a mental disability as a cause of legal incapacity is discriminatory and contrary to equality before the law doctrine,^20^ it is hard to see how any existing mental health legislation would be fully compliant with the CRPD.

### Future challenges

We did not find any reduction in the incidence of coercive community care and treatment after the introduction of the capacity-based criteria. Given the lack of other empirical studies on the topic, this calls for future research on outcomes of capacity-based mental health laws, and, more generally, on the impact of CRPD on the use of coercion in the provision of mental healthcare and treatment.^23^ There is clear evidence from the debate surrounding the CRPD that the conflict between paternalism and decision-making capacity, or phrased in another way: the conflict between need for treatment and recognition of patients’ right to make autonomous decisions (including bad decisions), is central to clinicians’ approach to assessments of decision-making competence.^3,4,21,22,24^ Opponents of the CRPD have articulated their concerns as follows: ʻIn the name of protecting all these people from discrimination, they [patients] would be free to destroy their own lives and ruin the lives of their loved ones'.^21^ In our view, such statements, as well as the general debate, calls for further empirical studies on the effect of capacity-based mental health legislation. Given identified challenges related to defining decision-making capacity,^16,17^ and the lack of empirical evidence on how clinicians assess it in practice, future research should also include the rationale and outcome of referrals and capacity assessments at different points of the CTO placement, as validation of capacity assessments in everyday clinical practice is strongly needed.

### Strengths and limitations

The strengths of the study include that we have complete data for all CTOs at two study sites and over the full study period. Further, by excluding the year the legal reform from the pre/post analysis we were able to capture a distinct, but temporary, ‘weeding-out’ practice in 2017, a practice that, to our knowledge, has not been described previously. A pre/post comparison based on the date the legal change came into force would not have identified this phenomenon and would have given an inaccurate picture of the impact of the legal change.^13^

Our data are restricted to what was recorded in patients’ medical files. The quality of the entries varied, and some values were missing. The significant increase in CTO termination rates should be interpreted with some caution because some patients had several CTO terminations, which could cause a degree of dependency in the data. Interviews with patients, decision-makers and family members could have added information on whether recognition of the patients’ will, preferences and autonomy changed after the legal reform.

Our analysis was limited to 2 years following the legal reform. We cannot rule out that a longer follow-up period could have captured changes in the use of CTOs as clinicians improve their skills to assess patients’ decision-making competence, and increased recognition of patient autonomy. This underlines the need for studies exploring long-term effects of legal reforms in the use of coercive interventions in clinical practice.

### Implications

The expected reduction in incidence rates and duration of new CTOs after the introduction of a capacity-based criterion in the NMHA was not confirmed in our study. CTO prevalence was significantly reduced after the reform because of a sharp increase in terminations of CTOs in the year the legal changes were passed and took effect. Given the many challenges related to defining and assessing decision-making capacity, studies validating such assessments in everyday clinical practice are urgently needed.

## Data Availability

The data that support the findings of this study are available on reasonable request from the first author. Data are not publicly available as they contain information that could compromise the privacy of included patients.

## Supplementary material

For supplementary material accompanying this paper visit http://doi.org/10.1192/bjo.2021.1073. click here to view supplementary material

## Appendix

### The criteria for involuntary care and treatment in the Norwegian Mental Health Act

**Chapter 3, Section 3-3. Administrative decisions regarding compulsory mental health care**

On the basis of information from the medical examination pursuant to section 3-1 and compulsory observation, if any, pursuant to section 3-2, the responsible mental health professional will assess whether the following conditions for compulsory mental health care are satisfied:

1. Voluntary mental healthcare has been tried, to no avail, or it is obviously pointless to try this.
2. The patient has been examined by two physicians, one of whom shall be independent of the responsible institution, cf. section 3-1.
3. The patient is suffering from a serious mental disorder and application of compulsory mental health care is necessary to prevent the person concerned from either
  - having the prospects of his or her health being restored or significantly improved considerably reduced, or it is highly probable that the condition of the person concerned will significantly deteriorate in the very near future,^a^ or
  - constituting an obvious and serious risk to his or her own life and health or those of others on account of his or her mental disorder.^b^
4. *The patient lacks capacity to consent cf. Patients’ Rights Act section 4.3. This criterion does not apply if there is obvious and serious risk to his or her own life or the life or health of others.*^c,d^
5. The institution is professionally and materially capable of offering the patient satisfactory treatment and care and is approved in accordance with section 3-5.
6. The patient has been given the opportunity to state his or her opinion, cf, section 3-9.
7. Even though the conditions of the Act are otherwise satisfied, compulsory mental health care may only be applied when, after an overall assessment, this clearly appears to be the best solution for the person concerned, unless he or she constitutes an obvious and serious risk to the life or health of others. When making the assessment, special emphasis shall be placed on how great a strain the compulsory intervention will entail for the person concerned.

Translation from Norwegian: https://app.uio.no/ub/ujur/oversatte-lover/cgi-bin/sok.cgi?dato=&nummer=&tittel=psykisk&type=LOV&S%F8k=S%F8k^d^^25^

- Commonly described as the ‘need for treatment criterion’.
- Commonly described as the ‘dangerousness criterion’.
- Commonly described as the ‘capacity criterion’.
- The English translation of the Act does not include the new ‘capacity criterion’. This has been translated to English by the authors.
